# Alcohol and cause-specific mortality in Russia: the Know Your Heart Study 2015–23

**DOI:** 10.1186/s12889-024-20674-8

**Published:** 2024-11-12

**Authors:** Nikita A. Mitkin, Tormod Brenn, Tatiana N. Unguryanu, Sofia Malyutina, Sarah Cook, Alexander V. Kudryavtsev

**Affiliations:** 1 Department of Community Medicine, UiT The Arctic University of Norway, Tromsø, N- 9037 Norway; 2 International Research Competence Centre, Northern State Medical University, Troitsky Ave., 51, Arkhangelsk, 163069 Russia; 3 Department of Hygiene and Medical Ecology, Northern State Medical University, Troitsky Ave., 51, Arkhangelsk, 163069 Russia; 4 Research Institute of Internal and Preventive Medicine, Branch of Institute of Cytology and Genetics, Siberian Branch of the Russian Academy of Sciences, Bogatkova st., 175/1, Novosibirsk, 630008 Russia; 5 Department of Therapy, Hematology and Transfusiology, Novosibirsk State Medical University, Krasny Ave., 52, Novosibirsk, 630091 Russia; 6 School of Public Health, Imperial College London, White City Campus, 80-92 Wood Lane, London, W12 0BZ UK

**Keywords:** Alcohol, Mortality, Cardiovascular diseases, Neoplasms, External causes, Risk factors, Russia

## Abstract

**Background:**

Alcohol-related mortality in Russia exceeds the world average and presents a critical public health concern. This study assesses the impact of alcohol consumption levels on mortality and investigates mortality predictors among Russians, including people treated for alcohol-related diagnoses (narcology patients).

**Methods:**

We examined 2629 men and women aged 35–69 years who participated in the Know Your Heart study (2015–17), Arkhangelsk, Russia. The participants were categorized into five drinking levels (non-drinking, low-risk, hazardous, harmful, narcology patients) and followed up using a regional mortality database. We used Cox proportional hazards regressions to analyze sociodemographic and cardiovascular biomarkers as mortality predictors among narcology patients and general population and to compare mortality risks across the five drinking levels.

**Results:**

During a median follow-up of 6.3 years, 223 (8.5%) participants died. Age- and sex-standardized all-cause mortality rates per 100,000 person-years were 1229 (95% CI: 691–1767) in non-drinking participants, 890 (95%CI: 684–1096) and 877 (95%CI: 428–1325) in low-risk and hazardous drinking participants, 2170 (95%CI: 276–4064) in those with harmful drinking, and 4757 (95%CI: 3384–6131) in narcology patients. The largest proportions of deaths were caused by cardiovascular diseases (37.2%), neoplasms (20.2%), and external causes (13.9%). Compared with low-risk drinkers, narcology patients had higher risks of death with hazard ratios of 3.23 (95%CI: 2.02–5.16) for all-cause mortality, 3.25 (95%CI: 1.52–6.92) for cardiovascular diseases, 9.36 (95%CI: 2.63–33.3) for external causes, and 7.79 (95%CI: 3.34–18.1) for other causes. Neoplasm-related mortality did not differ between groups. All-cause mortality in the general population had positive associations with smoking, waist-to-hip ratio, resting heart rate, systolic blood pressure, high-sensitivity C-reactive protein, and negative associations with left ventricular ejection fraction (LVEF) and higher education. These associations were substantially weaker and non-significant in narcology patients. Cardiovascular mortality in narcology patients was increased with higher education, while male sex, LVEF and N-terminal prohormone of brain natriuretic peptide had less impact compared to the general population sample.

**Conclusion:**

Narcology patients face markedly higher mortality risks—threefold from all causes and cardiovascular diseases, ninefold from external causes, and sevenfold from other causes. Compared with the general population, conventional mortality risk factors were less predictive of deaths in narcology patients.

**Supplementary Information:**

The online version contains supplementary material available at 10.1186/s12889-024-20674-8.

## Background

Alcohol consumption is a substantial risk factor for morbidity and mortality worldwide [1, 2], with approximately 3 million deaths (5.3%) attributed to alcohol in 2016 [3]. In the same year, alcohol emerged as the leading mortality risk factor for individuals aged 15–49, showing a higher mortality proportion among men (12.2%) compared with women (3.8%) [4]. Global per capita alcohol consumption has risen from 5.9 L to 6.5 L over the past three decades and is projected to reach 7.6 L by 2030 [5], which will increase the alcohol-related health burden [6, 7].

Russia stands among the highest alcohol-consuming countries and has about 20% higher prevalence of heavy episodic drinking than the global average [3, 8]. The country has historically had a specific structure of alcohol consumption with a predominance of hard liquor and binge drinking [9–12]. These habits significantly add to the high national burden of alcohol-related morbidity and mortality [13–17]. A striking example of such health losses is the mortality crisis during Russia’s late-1980s to early-2000s, where alcohol consumption was a primary mortality contributor among adult men and led to a reduction in life expectancy [18–20].

In 2016, Russian per capita alcohol consumption was estimated to be 11.7 L, surpassing the global average of 6.4 L [3], and caused 21.6% of all deaths in the country, double that of Europe [21]. In 2018, Russia recorded approximately 200,000 alcohol-related deaths [22], a quarter of which were directly attributed to alcohol, encompassing alcoholic cardiomyopathies, poisonings, and liver diseases. At the same time, 75% of these deaths were caused by cardiovascular disease, cancer, and other causes indirectly contributed to alcohol [23, 24].

According to previous studies, the relation between alcohol consumption and mortality is complex [25, 26] and may be characterized by a J-shaped curve [27–31]. Light and moderate drinkers tend to have a lower mortality risk compared to non-drinkers [28, 32, 33], while heavy and binge drinkers face substantially higher mortality rates [4, 34–36]. According the World Health Organization, people with alcohol use disorder (AUD) account for about 21% of the Russian adult population, which is four times the global prevalence (5.1%) [3]. Despite their relatively small numbers, the mortality risk in such groups can be 10 times higher than in the general population [37–39], being a source of excess mortality. A smaller but still significant excessive mortality risk was found among binge drinkers in the HAPIEE study in 4 Eastern European cohorts combined [40].

Targeted interventions have the potential to reduce mortality in such high-risk groups [41, 42], but are rare due to limited knowledge about predictors of death among heavy drinkers except for alcohol consumption itself [43–46]. Recent advances in eHealth systems in Russia offer new opportunities for prospective studies on alcohol-related mortality and its determinants [22, 47–50], enabling the integration of individual health data with mortality registries [51].

This study aims to estimate and compare mortality in Russians categorized by alcohol consumption levels followed for 6 years.

## Materials and methods

### Study sample

We used baseline data from the Know Your Heart (KYH) cross-sectional study conducted in Arkhangelsk, Russia, 2015–2017, described in detail by Cook et al. [52]. For this study, 2,629 KYH participants who signed informed consent for the access to medical records were followed up for cause-specific mortality until 30 April 2023. The sample included 2,357 men and women aged 35–69 years from the general population (main study participants) and 272 men and women of the same age treated for alcohol-related diagnoses (narcology patients).

The main study participants were sampled from Arkhangelsk residents with compulsory medical insurance using the depersonalized address list of the regional health insurance fund, supplemented by 5-year age band and sex information. Trained interviewers visited randomly selected addresses, identified target individuals by sex and age, and invited them to participate. Those who agreed underwent a baseline interview at home, followed by a health check at the polyclinic of the Northern State Medical University. The response rate for the baseline interview was 68%, and 96% of the interviewed participated in the health check.

Narcological patients were recruited from in-patients of Arkhangelsk Regional Psychiatric Hospital treated for alcohol-related diagnoses. Hospital staff invited patients to participate in the study at least one week after admission for detoxification, withdrawal management, or other acute alcohol-related conditions. According to the International Classification of Diseases, version 10 (ICD-10), the primary diagnoses of narcology patients were F10.3 Withdrawal state (54.4%), F10.4 Withdrawal state with delirium (21.7%), F10.1 Psychotic disorder (12.5%), F10.2 Dependence syndrome (5.5%), F10.8 Other mental and behavioral disorders (5.2%), and F10.5 Amnesic syndrome (0.7%). The response rate was 85%. Participants underwent a shortened version of the baseline interview at the hospital, followed by a health check at the polyclinic of the Northern State Medical University.

### Baseline data collection

The KYH baseline interview collected data on sociodemographic, behavioral and health characteristics and comprised a section with questions on alcohol consumption in the past 12 months. The questions covered types and amounts of alcoholic beverages consumed [9, 53] and included the Cut down, Annoyed, Guilty and Eye- opener (CAGE) questionnaire, which is, used for alcohol dependence screening [54–56]. This part also included questions on signs of harmful drinking: frequency of “zapoi” episodes (an alcohol drinking period of two or more days, during which a person is withdrawn from routine social life), hangover, excessive drunkenness, sleeping in clothes at night due to drunkenness, and failing to fulfill family or other obligations because of being drunk.

The health check was performed by trained medical staff and comprised a medical interview, a comprehensive examination of cardiovascular health, and biological sample collection. The medical interview included questions about medical history, treatment received, and the Alcohol Use Disorders Identification Test (AUDIT) test [57–59].

Cardiovascular health assessments included measurements of height (cm) using a Seca^®^ 217 stadiometer (Seca Limited), weight (kg) using a TANITA BC 418 body composition analyzer (TANITA, Europe GmbH), waist circumference (WC), and hip circumference (HC) in centimeters using a measuring tape (Seca^®^201; Seca Limited). Height, WC, and HC were measured twice, and the average of the two measurements was used in the analysis. Systolic and diastolic BP (SBP and DBP) and heart rate (HR) were measured at the brachial artery using an OMRON 705 IT automatic tonometer (OMRON Healthcare). SBP, DBP and HR were measured three times after five minutes of rest, with a 2-minute interval between measurements. The mean values of the second and third measurements were used in the analysis.

Blood samples were collected at least four hours post-meal, centrifuged, frozen at -80 °C, and transported to a laboratory in Moscow, where they were analyzed in a single batch at the end of the fieldwork. Laboratory personnel were blind to participants’ characteristics. Total cholesterol, low-density lipoprotein cholesterol (LDL-C), and triglyceride levels were assessed in serum by enzyme calorimetry, glycated hemoglobin (HbA1c) and high-sensitivity C-reactive protein (hCRP) – by immunoturbidimetric tests, Cystatin C – by particle-enhanced immunoturbidimetric test (AU 680; Chemistry System Beckman Coulter). Gamma-glutamyltransferase (GGT) levels were assessed using a kinetic color test. High-sensitivity troponin T (hsTnT) and brain natriuretic peptide (NT-proBNP) concentrations were assessed by the ECLIA electrochemiluminescent method (Cobas e411 Analyzer; Roche Diagnostics GmbH, Hitachi, Japan). The concentration of carbohydrate-deficient transferrin (CDT), a biomarker of chronic alcohol use, was determined by capillary electrophoresis (CAPILLARYS-2 automatic capillary electrophoresis system, Sebia S.A., France). GGT was measured in all study participants, whilst CDT analyzes were performed for a subsample of 1242 participants (976 from the KYH main study sample and 266 narcological patients).

### Exposure variable

We categorized the participants into five alcohol consumption levels based on their self-reported drinking behaviors in the past 12 months:

#### Non-drinking

Individuals reported no alcohol consumption.

#### Low-risk drinking

Participants consumed alcohol and had AUDIT scores below 8 and CAGE scores below 2.

#### Hazardous drinking

Participants consumed alcohol with AUDIT scores between 8 and 15, and/or CAGE scores between 2 and 3.

#### Harmful drinking

A participant met any of the following criteria: (a) AUDIT score of 16 or higher [57, 58, 60], (b) CAGE score of 4 [54, 55], (c) signs of harmful drinking pattern previously found to be highly predictive of mortality in Russia [9]: ≥1 episodes of “zapoi” (an alcohol drinking period of two or more days, during which a person is withdrawn from their routine social life), or ≥ 2 cases per week of hangover or excessive drunkenness or sleeping in clothes at night because of drunkenness or failing to fulfil family or social obligations because of alcohol use.

#### Narcology patients

People receiving inpatient treatment for alcohol-related diagnoses.

This categorization was applied in earlier KYH studies and was found valid by demonstrating the differences between the groups in annual volume of alcohol consumption, medical care seeking for alcohol-related issues, and blood biomarkers of excessive drinking (GGT and CDT) [61–63]. By utilizing multiple measures of alcohol exposure, this approach minimizes recall bias compared to a single measure like the frequency-quantity method [61, 64].

### Follow-up data collection

The KYH participants were linked to the Arkhangelsk Regional Mortality Database (ARMD) from the date of participation in KYH.

Data linkage between KYH and ARMD is regulated by agreements between NSMU and the Medical Information Analytical Center (MIAC) of the Arkhangelsk Regional Healthcare Ministry, ensuring confidentiality based on informed consents and legal/ethical approvals. Secure communication channels are used for data exchange. According to the confidentiality agreement, MIAC received a key file linking the KYH participants’ anonymous ID numbers to their personal identifiers. This enables MIAC to identify participants in the ARMD and provide mortality information to the KYH study team at NSMU with ID numbers only. Upon receipts of mortality data, the study team links them to KYH baseline data without participants’ personal information.

For this study, we used mortality data until 30 April 2023. For each death case, MIAC provided depersonalized death certificate data, including participant’s ID, date of death, immediate cause of death, related pathological conditions, underlying cause of death, external cause of death, and other contributing conditions according to ICD-10. Categorization of deaths by causes for this study was based on records of the underlying causes.

### Outcome variable

The outcome in this study was death from any cause and major causes of death, using data on the underlying cause of death. The major (most common) causes of death were defined according to chapters of the ICD-10, which were recorded for ≥ 10% of total deaths.

### Statistical analysis and covariates

Categorical variables were presented as counts and relative frequencies (percentage). Continuous variables were presented as means with 95% confidence intervals (CI) or medians with 25th and 75th percentiles (P25 and P75), depending on data distributions.

Health-related and laboratory parameters, adjusted for sex and age, were displayed as marginal mean or median values with 95% CI, which were assessed using analysis of covariance (ANCOVA) for parameters following a normal distribution, and quantile regression for those with skewed distribution. Mortality was estimated and presented as rates per 100,000 person-years, both crude and standardized by age and sex to the 2013 European Standard Population. The distribution of death by causes was presented as three major causes followed by three most contributing blocks of the corresponding ICD-10 chapters.

The associations between alcohol consumption levels with all-cause mortality and with mortality from major causes were estimated using multivariable Cox regression models, where study time was used as the timescale. At the first stage, we investigated mortality predictors in participants from the general population and in the narcology group, adjusting for sex and age. Between-group comparisons of the strength of the associations between mortality outcomes and the studied predictors were performed in sex- and age-adjusted Cox regression models repeated with pooled data through assessing interactions of the group-defining variable with a covariate of interest. Interactions were assessed by comparing models with and without interaction terms using likelihood ratio tests. The analyzed predictors included sociodemographic and behavioral characteristics (sex, age, higher education, marital status, smoking) and risk factors and biomarkers of cardiovascular disease (BMI, waist-to-hip index, resting HR, systolic and diastolic blood pressure, total cholesterol, low-density lipoproteins (LDL), triglycerides, glycated hemoglobin (HbA1c), high-sensitivity C-reactive protein (Hs-CRP), cystatin C, NT-proBNP, high-sensitivity troponin T (Hs-Troponin T), and left ventricle ejection fraction (LVEF), which were measured in the KYH study.

At the last stage, parameters that had associations (*p* < 0.2) with a mortality outcome in the general population sample and/or in the narcology group were adjusted for in the concluding multivariable regression analyses of the effects of alcohol consumption on all-cause mortality and mortality from major causes. The adjustments were initially performed for sociodemographic and behavioral parameters as potential confounders (Model A). At the second step (Model B), cardiovascular risk factors and biomarkers were adjusted for to assess their mediating roles. The handling, processing, and analyses of data was done using STATA 17.0 (StataCorp, USA, Texas, College Station).

## Results

### Baseline characteristics

The study cohort distribution across the five drinking levels was: non-drinking (8.9%, *N* = 236), low-risk drinking (63.0%, *N* = 1656), hazardous drinking (13.7%, *N* = 360), harmful drinking (4.0%, *N* = 105), and narcology patients (10.4%, *N* = 272) (Table 1).

There were higher proportions of men in the hazardous drinking (78.1%), harmful drinking (87.6%), and narcology (76.1%) categories, compared to low-risk drinking (30.2%) and non-drinking (46.2%). Median age had a decreasing trend from non-drinkers (57.9 years) to narcology patients (47.5 years) (*p* < 0.001).

Higher education was most common in low-risk drinking (39.3%), least common – in narcology group (9.6%) (*p* < 0.001). Hazardous drinking group had the highest proportion of married participants (67.8%), whereas in narcology group, the proportion was 20.7% (*p* < 0.001). Current smoking prevalence ranged from 22.0% in non-drinking participants to 82.0% in narcology patients (*p* < 0.001).

Median AUDIT and CAGE scores rose from 2 to 0 in low-risk drinking to 20 and 3 in narcology, respectively (*p* < 0.001). Harmful drinking patterns were exceptionally present in the harmful drinking (73.3%) and narcology groups (83.8%). Median CDT (%) and GGT (U/L) values increased from 0.54 to 21.8 in non-drinking participants to 1.64 and 58.9 in narcology patients, respectively, indicating increased alcohol exposure (*p* < 0.001).

**Table 1:** Alcohol use, sociodemographic and lifestyle characteristics by levels of alcohol consumption, adjusted for age and sex, the Know your heart study 2015–17

Study variables	Non-drinking	Low-risk drinking	Hazardous drinking	Harmful drinking	Narcology	*P* for trend
*N* (%)	236 (8.9)	1656 (63.0)	360 (13.7)	105 (4.0)	272 (10.4)	
Age, years, Me (P25; P75)	57.9 (49.1; 65.1)	54.7 (45.8; 62.7)	50.4 (43.8; 58.8)	51.9 (43.8; 58.7)	47.5 (40.9; 54.5)	< 0.001^a^
Male sex, N (%)	109 (46.2)	500 (30.2)	281 (78.1)	92 (87.6)	207 (76.1)	< 0.001^b^
Higher education, N (%)	64 (27.1)	651 (39.3)	120 (33.3)	19 (18.1)	26 (9.6)	< 0.001^b^
Married, N (%)	143 (60.6)	1,015 (61.3)	244 (67.8)	56 (53.3)	56 (20.7)	< 0.001^b^
Current smoker, N (%)	52 (22.0)	290 (17.5)	138 (38.3)	68 (64.8)	223 (82.0)	< 0.001^b^
Abstainers ^c^, N (%)	236 (100.0)	/	/	/	/	/
AUDIT score, Me (P25; P75)	0 (0; 0)	2 (1; 4)	8 (6; 10)	13 (8; 16)	20 (15; 27)	< 0.001^a^
CAGE score, Me (P25; P75)	0 (0; 0)	0 (0; 0)	2 (1; 2)	3 (2; 4)	3 (3; 4)	< 0.001^a^
Harmful drinking pattern ^d^, N (%)	/	/	/	77 (73.3)	228 (83.8)	< 0.001^b^
CDT*, %, Me (P25; P75)	0.54 (0.47; 0.71)	0.74 (0.57; 1.01)	0.84 (0.67; 1.21)	0.94 (0.67; 2.71)	1.64 (0.97; 2.51)	< 0.001^a^
GGT, U/L, Me (P25; P75)	21.8 (15.4; 33.6)	24.1 (17.1; 35.9)	31.6 (20.8; 54.4)	36.6 (25.2; 61.2)	58.9 (34.4; 119.5)	< 0.001^a^

^a^ p-values for trends from quantile regression; ^b^ p-values for trends from logistic regression

^c^ Abstainers are those who reported no alcohol use in the past 12 months

^d^ Harmful drinking was defined as reporting one of the following: ≥1 episodes of “zapoi” (an alcohol drinking period of two or more days, during which a person falls out of a routine social life) in the past 12 months, ≥ 2 times per week cases of hangover and/or excessive drunkenness and/or sleeping in clothes at night because of drunkenness and/or failing to fulfil family or other obligations because of alcohol in the past 12 months

Definitions: AUDIT indicates Alcohol Use Disorders Identification Test; CAGE—The Cutting down, Annoyance by criticism, Guilty feeling, Eye-opener Test; CDT—carbohydrate-deficient transferrin; GGT—gamma-glutamyl transferase

* CDT data were available for 1242 KYH participants (74 in non-drinking group, 446 in low-risk drinkers, in 359 hazardous drinking group, 97 in harmful drinking group, 266 in narcology patients)

### Baseline health-related and laboratory parameters

The mean BMI was lowest in narcology (24.9 kg/m^2^) and highest among hazardous drinking (28.3 kg/m^2^) participants, who also had a higher mean WHR (0.90) compared to other groups (0.88–0.89) (Table 2). The lowest mean resting heart rate was in non-drinking (71.6) and highest in narcology patients (76.8). The mean SBP and DBP peaked in hazardous drinking group (135.2 and 86.2 mmHg) and were lowest in narcology patients (125.6 and 82.2 mmHg).

Means of total cholesterol and LDL were elevated in hazardous drinking participants (5.6 and 3.8 mmol/L) and the lowest in narcology group (5.1 and 3.3 mmol/L). In contrast, medians of Cystatin C (mg/L) and NT-proBNP (pg/mL) were lowest in hazardous drinking group (0.85 and 87.9) and highest in narcology patients (0.92 and 124.8, respectively). There were no significant between-group differences in HbA1c and hs-TroponinT. Triglycerides (mmol/L) and Hs-CRP (mg/L) were significantly higher in narcology patients (medians: 1.34 and 3.30, respectively) compared to other groups. LVEF was the highest in participants with hazardous drinking (57.1%) and the lowest in narcology group (55.6%).

**Table 2:** Health-related and laboratory parameters by levels of alcohol consumption, adjusted for age and sex, the Know your heart study 2015–17

Parameters	Non- drinking	Low-risk drinking	Hazardous drinking	Harmful drinking	Narcology	*P* value
*N* (%)	236 (8.9)	1656 (63.0)	360 (13.7)	105 (4.0)	272 (10.4)	
	Mean (95%CI)					
BMI ^a^, kg/m^2^	27.3 (26.6–27.9)	27.7 (27.5–28.0)	28.3 (27.7–28.8)	26.3 (25.2–27.3)	24.9 (24.3–25.6)	< 0.001
WHR ^a^	0.88 (0.87–0.89)	0.88 (0.87–0.88)	0.90 (0.90–0.91)	0.89 (0.88–0.91)	0.88 (0.87–0.89)	< 0.001
Resting heart rate ^a^, beats/min	71.6 (70.1–73.1)	73.2 (72.6–73.7)	73.6 (72.4–74.8)	76.0 (73.8–78.2)	76.8 (75.4–78.2)	< 0.001
SBP ^a^, mm Hg	129.0 (126.7–131.4)	131.8 (130.9–132.7)	135.2 (133.3–137.2)	132.8 (129.3–136.4)	125.6 (123.3–127.8)	< 0.001
DBP ^a^, mm Hg	80.5 (79.1–81.9)	83.3 (82.7–83.8)	86.2 (85.0–87.4)	84.2 (82.0–86.3)	82.2 (80.8–83.5)	< 0.001
Total cholesterol ^a^, mmol/L,	5.27 (5.14–5.41)	5.39 (5.33–5.44)	5.58 (5.46–5.69)	5.29 (5.08–5.50)	5.06 (4.93–5.20)	< 0.001
LDL ^a^, mmol/L	3.62 (3.50–3.73)	3.64 (3.60–3.68)	3.76 (3.67–3.86)	3.46 (3.29–3.63)	3.33 (3.22–3.43)	< 0.001
LVEF ^a^, %	56.3 (55.5–57.1)	56.6 (56.3–56.9)	57.1 (56.4–57.8)	57.0 (55.8–58.2)	55.6 (54.9–56.4)	0.039
	Median (95%CI)					
TG ^b^, mmol/L	1.16 (1.06–1.26)	1.21 (1.16–1.25)	1.29 (1.19–1.38)	1.18 (1.00–1.36)	1.34 (1.28–1.40)	0.001
HbA1c ^a^, %	5.54 (5.45–5.64)	5.53 (5.50–5.57)	5.56 (5.48–5.64)	5.50 (5.35–5.64)	5.61 (5.52–5.70)	0.591
hs-CRP ^b^, mg/L	1.37 (1.14–1.61)	1.56 (1.46–1.66)	1.77 (1.55–1.98)	1.85 (1.46–2.25)	3.30 (2.82–3.78)	< 0.001
Cystatin C ^b^, mg/L	0.90 (0.88–0.92)	0.87 (0.86–0.87)	0.85 (0.84–0.87)	0.88 (0.85–0.91)	0.92 (0.89–0.94)	0.007
NTpBNP ^b^, pg/mL	101.3 (89.1–113.6)	86.2 (81.8–90.6)	87.9 (81.4–94.5)	111.4 (91.8–131.0)	124.8 (111.4–138.1)	< 0.001
hs-TpT ^b^, pg/L	6.63 (6.26–7.01)	6.53 (6.36–6.70)	6.63 (6.29–6.97)	6.47 (5.93–7.01)	6.91 (6.36–7.45)	0.591

^a^ Means with 95%CI and p-values via ANCOVA, ^b^ Medians with 95%CI and p-values via quantile regression

Definitions–WHR, waist-to-hip ratio, SBP, systolic blood pressure; DBP, diastolic blood pressure; LDL, low-density lipoprotein; TG, triglycerides; HbA1c, glycated hemoglobin; hs-CRP, high-sensitivity C-reactive protein, NTpBNP, N-terminal prohormone of brain natriuretic peptide; hs-TpT. high sensitivity Troponin T; LVEF, left ventricular ejection fraction

### Mortality outcomes

Over a median follow-up of 6.3 years (IQR–6.0 to 7.0 years), 223 participants (8.5%) died (Table 3). Mortality proportions were notably higher in harmful drinking (15.2%) and narcology groups (26.8%) compared to non-drinking (9.8%), low-risk (5.3%), and hazardous drinking groups (6.4%). The overall median age at death was 62 years, with harmful drinking and narcology participants dieing younger (60 and 55 years, respectively) than other groups (65–67 years). Relative to low-risk drinking participants, age and sex-standardized mortality rates per 100,000 person-years was elevated in non-drinking group (1229), lowest in low-risk drinking (890) and hazardous drinking participants (877), and highest in narcology (4757) and harmful drinking groups (2170).

Cardiovascular diseases (ICD-10: I00–I99) were predominant causes of death (37.2%) across groups, accounting for 34.3% deaths in narcology to 43.8% in harmful drinking participants. Within this class of causes, ischemic heart diseases (I20–I25) constituted the majority, especially among non-drinkers (34.8%) but accounted for a smaller proportion in narcology patients (16.4%). Other heart diseases (I30–I52) accounted for 12.5% of deaths in harmful drinking and 12.3% in narcology.

Neoplasms (C00–D48) were the second major cause of death (20.2%), with the highest proportion of deaths among low-risk drinkers (31.8%) and the lowest in narcology patients (5.5%). Deaths from digestive system neoplasms (C15–C26) were most frequent in hazardous (17.4%) and low-risk drinkers (15.9%), but rare in narcology patients (1.4%).

External causes (V01–Y98) were the third major cause of death (13.9%), accounting for higher proportions of deaths in harmful drinking (25.0%) and narcology patients (27.4%) versus other groups (4.4–5.7%), with accidental poisoning (X40–X49) and intentional self-harm (Y60–X84) being the leading causes within the class.

Other causes of death are shown in Supplementary Table 1. Due to small numbers of deaths in the other categories we focus here on the three most common causes of death in the sample.

**Table 3:** Fatal outcomes by levels of alcohol consumption, the Know your heart study 2015–17

Fatal outcome parameters	Non-drinking	Low-risk	Hazardous drinking	Harmful drinking	Narcology	Total
*N*	236	1656	360	105	272	2629
Follow-up duration, person-years(p-y)	1500	10,737	2301	650	1379	16,567
Deaths, N (%)	23 (9.8)	88 (5.3)	23 (6.4)	16 (15.2)	73 (26.8)	223 (8.5)
Age at death, Me (IQR)	66 (60–70)	67 (61–71)	65 (54–73)	60 (53–64)	55 (47–60)	62 (53–68)
Crude all-cause mortality rate per 100 000 p-y (95% CI)	1533 (1019; 2308)	819 (665; 1010)	998 (664; 1503)	2459 (1507; 4015)	5293 (4209; 6659)	1345 (1180; 1534)
Age- and sex-standardized all-cause mortality rate* per 100 000 p-y (95% CI)	1229 (691; 1767)	890 (684; 1096)	877 (428; 1325)	2170 (276; 4064)	4757 (3384; 6131)	1374 (1191; 1558)
Major causes of death ^a^, N (%)						
I00-I99. Cardiovascular	10 (43.5)	32 (36.4)	10 (43.5)	7 (43.8)	25 (34.3)	84 (37.2)
I20-I25. Ischemic heart diseases	8 (34.8)	21 (23.9)	7 (30.4)	< 5^d^	12 (16.4)	51 (22.9)
I30-I52. Other heart diseases	/	5 (5.7)	< 5^d^	< 5^d^	9 (12.3)	16 (7.2)
I60-I69. Cerebrovascular diseases	< 5^d^	< 5^d^	< 5^d^	< 5^d^	< 5^d^	13 (5.8)
C00-D48. Neoplasms	6 (26.1)	28 (31.8)	5 (21.7)	2 (12.5)	4 (5.5)	45 (20.2)
C15-C26. Digestive system	< 5^d^	14 (15.9)	< 5^d^	< 5^d^	< 5^d^	23 (10.3)
C30-C39. Respiratory system	< 5^d^	< 5^d^	< 5^d^	/	< 5^d^	8 (3.6)
C50. Breast cancer	/	< 5^d^	/	/	/	< 5^d^
V01-Y98. External causes	1 (4.4)	5 (5.7)	1 (4.4)	4 (25.0)	20 (27.4)	31 (13.9)
X40-X49. Accidental poisoning	/	< 5^d^	/	< 5^d^	5 (6.8)	8 (3.6)
Y10-Y34. Undetermined intent	/	< 5^d^	/	/	< 5^d^	6 (2.7)
Y60-X84. Intentional self-harm	< 5^d^	/	/	/	< 5^d^	5 (2.2)
Other causes ^b^	6 (26.1)	23 (26.1)	7 (30.4)	< 5^d^	24 (32.9)	63 (28.3)

* Age- and sex-standardized to European Standard Population 2013

^a^ Shown as ICD-10 chapters with a contribution of ≥ 10% to total deaths and three most contributing ICD-10 blocks within a chapter

^b^ Other causes included following ICD-10 chapters–Certain infectious and parasitic diseases (A00-B99), Endocrine, nutritional and metabolic diseases (E00-E90), Diseases of the nervous system (G00-G99), Diseases of the respiratory system (J00-J99), Diseases of the digestive system (K00-K93), Diseases of the genitourinary system (N00-N99), Congenital malformations, deformations and chromosomal abnormalities (Q00-Q99), Symptoms, signs and abnormal clinical and laboratory findings, not elsewhere classified (R00-R99)

^d^ Numbers redacted due to cell counts less than 5 to preserve data anonymity

### Mortality predictors in general population and narcology patients

The KYH main study participants (including all defined as non-drinking, low-risk, hazardous, and harmful drinking groups in this study) and narcology patients showed distinct differences in predictors of all-cause mortality (Table 4). Higher education was a protective factor in the main study but not in narcology patients. Smoking increased mortality risk in the main study but had no effect in narcology patients. The impact of NT-proBNP was less pronounced in narcology patients compared to the main study. Other significant predictors in the main study but not in narcology patients included WHR, resting HR, SBP, Hs-CRP, and LVEF.

For circulatory disease mortality, male sex posed a higher risk in the main study but not in narcology patients. Higher education was a protective factor in the main study but increased the risk in narcology patients. Smoking was a risk factor in the main study group but not in narcology patients. BMI significantly impacted circulatory disease mortality only in narcology patients. NT-proBNP had greater impact in the main study than in narcology. Increased LVEF reduced cardiovascular mortality risks in the main study, with no effect on narcology patients.

For deaths from neoplasms, there were no significant differences between the main study and narcology patients in effects of the studied characteristics, which can be explained by a small power due to the limited number of cases.

In the context of mortality from external causes, a lower BMI increased risk in the main study but had no effect in narcology patients.

In the main study, the risk of death from other causes increased per unit increase in BMI, WHR, HbA1c, NT-proBNP, Hs-Troponin T, resting HR, and Hs-CRP. Among these characteristics, only resting HR and Hs-CRP showed significant but inverse effects in narcology patients.

**Table 4:** Associations of sociodemographic, behavioral, and health characteristics associated with all-cause and cause-specific mortality among the main study and narcological patients, adjusted for age and sex. The Know your heart study 2015–17

	Any death (*N* = 223)		Death from circulatory disease (*N* = 84)		Death from neoplasm (*N* = 45)		Death from external cause (*N* = 31)		Death from other cause (*N* = 63)	
	Main study	Narcology	Main study	Narcology	Main study	Narcology	Main study	Narcology	Main study	Narcology
	HR (95%CI)		HR (95%CI)		HR (95%CI)		HR (95%CI)		HR (95%CI)	
Age, years	1.08 (1.06–1.10)	1.03 (1.00–1.06)	1.07 (1.04–1.10)	1.06 (1.01–1.10)	1.13 (1.08–1.19)	1.11 (0.99–1.24)	1.00 (0.94–1.06)	0.99 (0.93–1.04)	1.08 (1.04–1.12)	1.03 (0.98–1.08)
Sex, male	3.06 (2.18–4.31)	1.69 (0.91–3.13)	4.72 (2.59–8.60)i	1.32 (0.49–3.51)i	2.38 (1.27–4.46)	0.99 (0.10–9.55)	3.87 (1.03–14.6)	6.40 (0.86–47.9)	2.15 (1.14–4.08)	1.27 (0.47–3.40)
Higher education	0.45 (0.30–0.68)i	1.33 (0.66–2.69)i	0.32 (0.15–0.67)i	2.22 (0.83–5.94)i	0.52 (0.24–1.12)	2.71 (0.28–26.5)	0.40 (0.09–1.89)	1.26 (0.29–5.47)	0.62 (0.29–1.31)	0.41 (0.05–3.03)
Married	0.56 (0.40–0.80)	0.79 (0.43–1.44)	0.45 (0.26–0.79)	1.06 (0.42–2.68)	0.88 (0.43–1.80)	2.89 (0.39–21.3)	1.07 (0.27–4.27)	0.47 (0.11–2.04)	0.44 (0.22–0.88)	0.51 (0.15–1.75)
Smoking	2.88 (2.05–4.05)i	0.99 (0.54–1.82)i	2.34 (1.37–4.00)i	0.78 (0.30–2.01)i	3.78 (1.96–7.31)	1.45 (0.12–16.9)	7.65 (1.93–30.4)	0.97 (0.27–3.49)	2.38 (1.20–4.72)	1.38 (0.44–4.32)
BMI, kg/m2	1.01 (0.98–1.04)	1.01 (0.95–1.07)	0.99 (0.94–1.05)i	1.11 (1.01–1.22)i	1.02 (0.97–1.08)	0.96 (0.74–1.25)	0.74 (0.62–0.88)i	0.93 (0.81–1.07)i	1.05 (1.00–1.11)i	0.93 (0.83–1.05)i
WHR, %	1.03 (1.01–1.06)i	1.00 (0.96–1.04)i	1.01 (0.97–1.05)	1.03 (0.96–1.10)	1.04 (0.99–1.09)	0.91 (0.77–1.07)	0.98 (0.90–1.08)	1.00 (0.93–1.07)	1.07 (1.03–1.12)i	0.98 (0.91–1.05)i
Resting HR, beats/min	1.02 (1.01–1.03)i	0.99 (0.97–1.01)i	1.03 (1.01–1.05)	1.01 (0.98–1.04)	1.01 (0.99–1.04)	1.00 (0.93–1.09)	1.01 (0.96–1.06)	0.99 (0.96–1.02)	1.02 (1.00–1.05)i	0.98 (0.94–1.01)i
SBP, Hg mm	1.01 (1.00–1.01)i	1.00 (0.99–1.02)i	1.00 (0.99–1.02)	1.01 (0.98–1.03)	1.00 (0.98–1.01)	0.96 (0.90–1.02)	1.03 (1.00–1.06)	0.99 (0.97–1.02)	1.01 (1.00–1.03)	1.01 (0.99–1.04)
DBP, Hg mm	1.01 (0.99–1.02)	0.99 (0.97–1.01)	1.01 (0.99–1.04)	1.02 (0.98–1.05)	0.99 (0.96–1.02)	0.93 (0.84–1.02)	0.99 (0.94–1.05)	0.98 (0.94–1.02)	1.01 (0.99–1.04)	0.99 (0.95–1.03)
Total chol., mmol/L	0.90 (0.78–1.04)	0.99 (0.77–1.27)	0.92 (0.73–1.17)	1.00 (0.65–1.53)	0.91 (0.69–1.19)	1.66 (0.62–4.47)	0.57 (0.31–1.05)	1.02 (0.61–1.69)	0.96 (0.73–1.28)	0.88 (0.56–1.37)
LDL-C, mmol/L	0.88 (0.73–1.05)	1.09 (0.81–1.47)	0.88 (0.66–1.18)	1.13 (0.68–1.87)	0.97 (0.70–1.35)	2.76 (0.90–8.49)	0.40 (0.19–0.84)	0.97 (0.52–1.79)	0.94 (0.66–1.33)	0.96 (0.56–1.63)
Triglycerides, mmol/L	1.10 (0.98–1.24)	1.03 (0.67–1.59)	1.02 (0.82–1.26)	0.77 (0.35–1.72)	1.22 (1.02–1.46)	0.90 (0.13–6.35)	0.36 (0.11–1.17)	1.13 (0.50–2.56)	1.18 (0.97–1.43)	1.32 (0.65–2.66)
HbA1, %	1.40 (1.24–1.57)	1.24 (0.95–1.63)	1.27 (1.02–1.58)	1.50 (1.07–2.12)	1.13 (0.84–1.53)	1.43 (0.44–4.61)	0.45 (0.17–1.21)	0.94 (0.42–2.08)	1.77 (1.52–2.05)i	1.10 (0.61–1.99)i
Hs-CRP, mg/L	1.02 (1.01–1.03)i	1.00 (0.98–1.02)i	1.01 (1.00–1.03)	0.99 (0.96–1.03)	1.02 (1.01–1.03)	0.96 (0.82–1.12)	1.02 (0.98–1.06)	1.02 (1.00–1.03)	1.02 (1.00–1.03)i	0.94 (0.87–1.02)i
Cystatin C, mg/L	1.47 (1.24–1.74)	2.51 (0.77–8.18)	1.31 (0.93–1.87)	6.75 (1.31–34.8)	1.37 (0.95–1.97)	12.5 (0.35–449)	1.17 (0.31–4.45)	0.11 (0.00–2.88)	1.72 (1.36–2.18)	2.24 (0.27–18.9)
NT-proBNP, pg/mL	1.97 (1.71–2.27)i	1.22 (1.00–1.49)i	2.26 (1.85–2.75)i	1.38 (0.98–1.94)i	1.40 (1.03–1.90)	3.56 (1.42–8.95)	2.06 (1.34–3.16)	1.02 (0.69–1.50)	1.99 (1.48–2.68)i	1.04 (0.73–1.49)i
Hs-Troponin T, ng/L	1.04 (1.02–1.05)	1.02 (0.99–1.06)	1.04 (1.03–1.05)	1.04 (0.99–1.10)	1.03 (1.01–1.06)	1.02 (0.88–1.19)	0.97 (0.81–1.16)	1.06 (0.99–1.13)	1.03 (1.00–1.06)i	0.94 (0.84–1.05)i
LVEF, %	0.94 (0.92–0.97)i	1.00 (0.96–1.05)i	0.91 (0.88–0.94)i	1.02 (0.95–1.09)i	0.97 (0.92–1.02)	0.86 (0.73–1.02)	0.96 (0.87–1.06)	1.05 (0.96–1.14)	1.00 (0.94–1.06)	0.98 (0.92–1.05)

Colored black if *p* < 0.2; **ln-transformed; ^i^ Significant interaction of variable with study group

### Risk of death by level of alcohol use

With adjustment for sociodemographic and behavioral factors (Model A), narcology patients demonstrated a significantly higher all-cause mortality risk (HR 3.79) relative to low-risk drinking participants (Table 5). Similar effect of being a narcology patient was observed for cardiovascular deaths (HR 3.29). For external causes of death, harmful drinking and narcology groups had higher risks than in low-risk drinking group (HRs 4.97 and 11.9, respectively). For other causes of death, a higher risk was observed among narcology patients (HR 5.73) relative to the low-risk category. Neoplasm-related mortality risk showed no significant differences across alcohol consumption levels.

Further adjustment for cardiovascular risk factors and biomarkers (Model B), which were considered as possible mediators, resulted in similar HRs for narcology patients. Relative to low-risk group, narcology patients maintained elevated risks of all-cause mortality (HR 3.23), cardiovascular death (HR 3.25), death from external causes (HR 9.36), and other causes of death (HR 7.79). The risk of death from external causes in harmful drinkers became non-significant in Model B (HR 4.07) although still high, and the risk for neoplasm-related deaths remained non-significant across all groups. In the non-drinking group, point estimates of HRs were lower generally in Model B compared to Model A after adjusting for cardiovascular risk factors and biomarkers. However, the same adjustments resulted it increased HR points estimates in hazardous and harmful drinking groups.

**Table 5:** Associations of alcohol consumption levels with all-cause and cause-specific mortality, HR (95% CI). The Know your heart study 2015–17

Cause of death	Non- drinking	Low-risk drinking	Hazardous drinking	Harmful drinking	Narcology
*N* (%)	236 (9.0)	1656 (63.0)	360 (13.7)	105 (4.0)	272 (10.3)
Model A^a^					
Any cause of death ^(1)^	1.35 (0.85–2.14)	Ref.	0.86 (0.53–1.39)	1.48 (0.84–2.59)	**3.79 (2.51–5.73)**
Cardiovascular ^(2)^	1.57 (0.77–3.20)	Ref.	0.95 (0.46–1.99)	1.65 (0.70–3.92)	**3.29 (1.66–6.52)**
Neoplasms ^(3)^	1.07 (0.44–2.60)	Ref.	0.67 (0.25–1.80)	0.66 (0.15–2.89)	1.06 (0.34–3.34)
External causes ^(4)^	1.05 (0.12–9.09)	Ref.	0.48 (0.05–4.22)	**4.97 (1.21–20.5)**	**11.9 (3.71–38.2)**
Other causes ^(5)^	1.47 (0.60–3.62)	Ref.	1.22 (0.50–2.96)	1.37 (0.39–4.84)	**5.73 (2.66–12.4)**
Model B^b^					
Any cause of death ^(i)^	1.19 (0.70–2.04)	Ref.	1.05 (0.63–1.77)	1.75 (0.95–3.22)	**3.23 (2.02–5.16)**
Cardiovascular ^(ii)^	1.64 (0.57–2.80)	Ref.	1.26 (0.57–2.80)	2.05 (0.80–5.26)	**3.25 (1.52–6.92)**
Neoplasms ^(iii)^	0.51 (0.15–1.76)	Ref.	0.56 (0.18–1.76)	0.79 (0.18–3.51)	0.50 (0.12–2.07)
External causes ^(iv)^	1.10 (0.12–9.78)	Ref.	0.50 (0.05–4.54)	4.07 (0.92–18.1)	**9.36 (2.63–33.3)**
Other causes ^(v)^	1.63 (0.61–4.37)	Ref.	1.42 (0.56–3.56)	1.64 (0.45–5.97)	**7.79 (3.34–18.1)**

^a^ Model A was adjusted for– [1] age, sex, higher education, marital status, smoking status for any cause of death and deaths from cardiovascular diseases; [2] age, sex, higher education, smoking status for deaths from neoplasms; [3] sex and smoking status for deaths from external causes; [4] age, sex, marital status, smoking status for deaths from other causes

^b^ Model B was adjusted as per Model A plus–(i) WHR, HR, SBP, total cholesterol, LDL, triglycerides, HbA1c, hs-CRP, Cystatin C, NT-ProBNP, hs-Troponin T and LVEF for any cause of death; (ii) BMI, HR, HbA1c, hs-CRP, Cystatin C, NT-ProBNP, hs-Troponin T, LVEF for cardiovascular deaths; (iii) WHR, SBP, LDL, triglycerides, hs-CRP, Cystatin C, NT-ProBNP, hs-Troponin T and LVEF for deaths from neoplasms; (iv) BMI, SBP, LDL, total cholesterol, triglycerides, HbA1c, HbA1c, hs-CRP and NT-proBNP, hs-Troponin T for deaths from external causes; (v) BMI, WHR, HR, SBP, triglycerides, HbA1c, hs-CRP, Cystatin C, NT-ProBNP, hs-Troponin T for deaths from other causes

Other causes included following ICD-10 Chapters–Certain infectious and parasitic diseases (A00-B99), Endocrine, nutritional and metabolic diseases (E00-E90), Diseases of the nervous system (G00-G99), Diseases of the respiratory system (J00-J99), Diseases of the digestive system (K00-K93), Diseases of the genitourinary system (N00-N99), Congenital malformations, deformations and chromosomal abnormalities (Q00-Q99), Symptoms, signs and abnormal clinical and laboratory findings, not elsewhere classified (R00-R99)

## Discussion

In this prospective cohort study, we explored the effect of alcohol consumption level on all-cause and cause-specific mortality in a Russian adult cohort followed up for over six years. To our knowledge, this is the first Russian study linking mortality register records to survey data covering both general population and in-patients treated for alcohol-related diagnoses.

Our study demonstrated three times higher mortality risks in narcology patients compared to those with low-risk drinking. This finding is consistent with existing literature. For instance, Rivas et al. reported a crude mortality rate of 3.3 per 100 person-years among middle-aged Spanish in-patients with alcohol use disorders (AUD) [44]. Abdul-Rahman et al. observed a threefold increase in mortality in individuals with AUD, with cirrhosis and external causes as primary death reasons [65]. In a 40-years prospective cohort study, Kendler et al. revealed a nearly sixfold increase in all-cause mortality in people with AUD [39]. Roerecke and Rehm’s meta-analyses echoed these findings, showing a similar increase in mortality risks and a shift in the mortality profile toward external causes and mental disorders in populations with AUD [37, 41].

Our findings on higher mortality rates in harmful drinking group in the general population were in line with the results of Plunk et al. and Ricci et al., who reported a doubled mortality risks among heavy drinkers [35, 66]. This also aligns with findings of Bobak et al. in a Russian cohort, showing a comparable rise in mortality risk among binge and risky drinkers [25]. Similarly, in another Russian cohort, the risk of all-cause and cardiovascular death increased 1.6 and 2 times among frequent heavy drinkers [34]. In a large Eastern European cohort study, the authors reported a rise in mortality by 1.23 for all-cause, 1.38 for cardiovascular and 2.03 times for alcohol-related causes among men drinking > 60 g ethanol per day [40]. The variability in mortality across studies can be attributed to differences in sample characteristics, alcohol exposure measurements, definitions and classifications used, sociocultural contexts, and regional drinking patterns [9, 11, 67]. In our analysis, the marginally significant higher mortality risk in the harmful drinking group compared to low-risk drinkers could be attributed to the small group size and death count (*N* = 16). Despite this, our results support that prolonged excessive alcohol consumption markedly raises mortality risk.

Our study illustrated shifts in mortality profiles linked to different levels of alcohol consumption. Cardiovascular diseases remained the primary cause of death across all groups, but secondary causes varied. In non-drinking, low-risk, and hazardous drinking groups the second-leading mortality cause were neoplasms, whereas harmful drinking and narcology participants faced higher mortality from external causes like accidental poisoning and self-harm. This shift from health-related mortality causes in lower alcohol consumption levels to more external and behavioral causes in higher consumption groups aligns with changes in neurological pathways induced by alcohol [4, 41]. Alcohol’s influence on gamma-aminobutyric acid and glutamate pathways increases psychiatric disorders such as depression and anxiety [68] and causing alcoholic ketoacidosis due to poor nutritional status [69]. These effects impair judgment and motor coordination, raising risks of accidents, injuries, and self-harm [68, 70].

Our results align with previous studies showing higher cardiovascular mortality among people with AUD [41, 65, 71]. Research identifies alcohol as a factor in endothelial dysfunction, a precursor to atherosclerosis, and in oxidative stress, which damages cells [72]. In addition, alcohol increases myocardial wall stress, leading to rhythm disturbances and atrial fibrillation [73], escalating the risk for alcoholic cardiomyopathy, ischemic heart disease, heart failure, and stroke [74]. In our analyses, we attempted to explain the effects of alcohol on all-cause and cause-specific mortality by adjusting for biomarkers of cardiovascular disease (resting HR, systolic and diastolic blood pressure, total cholesterol, LDL, triglycerides, HbA1c, Hs-CRP, cystatin C, NT-proBNP, Hs-Troponin T, and LVEF) as potential mediators of the alcohol effects. However, these adjustments attenuated the alcohol-associated HRs only marginally. This potentially indicates independent harmful effect of chronic severe drinking on the circulatory system and shows the need to go beyond conventional cardiovascular risk management in this high-risk population.

Contrary to previous research [39, 71, 75], we did not find a significant association between alcohol consumption and neoplasm mortality, despite the well-documented carcinogenic effects of alcohol [76]. Similarly, while alcohol is known to cause hepatic inflammation [77], our study showed a lesser impact of digestive diseases on mortality in harmful drinking participants and narcology patients than reported previously [41, 65]. This discrepancy may be attributed to the younger age at death in these participants, precluding the development of cancer. The follow up duration might also not have been sufficient to capture the full impact of alcohol on cancer and digestive mortality, especially considering the relatively small size of these groups.

Our observation of increased mortality rates among non-drinking participants aligns with previous research but requires interpretation with caution [28]. Previous studies, such as those by Ding et al., Xi et al., and Stewart et al., have suggested a protective effect of moderate alcohol consumption in diverse populations including British cardiovascular patients and US adults with chronic diseases [32, 71, 78]. Ma et al. further linked regular drinking to lower mortality, regardless of the amount consumed [26]. These results, however, might be influenced by the “sick quitter” effect, where cessation of alcohol consumption is due to health issues, as indicated in studies excluding former drinkers and those with existing morbidities [27, 36, 75]. Supporting this, Ortolá et al. found no significant benefit from light-to-moderate drinking among older adults in Spain [79], and Jankhotkaew’s 30-year study in Thailand showed a linear increase in mortality with alcohol use [80]. In addition, a meta-analysis of 87 studies by Stockwell et al. concluded that moderate drinking had no mortality advantage over lifetime abstinence or occasional use [81]. These findings suggest that the higher mortality rates observed among non-drinkers in our study might reflect the underlying health characteristics, which explain the abstinence, rather than being a consequence of abstaining from alcohol.

In our study, hazardous and low-risk drinkers showed similar mortality rates, despite distinct drinking patterns. This finding contrasts with prior research linking heavy drinking (AUDIT scores ≥ 8) to increased mortality risks than in moderate drinkers [42, 82]. The observed discrepancy may be explained by our definition of hazardous drinking, narrowly defined by AUDIT scores of 8–15, represents a specific subset of the broader category of heavy drinkers examined in previous studies. The observed mortality parity between hazardous and low-risk drinkers might be also due to health selection, where individuals with initially better health engage in hazardous drinking without immediate consequences. However, this equal risk could change over time, suggesting that the long-term impacts of hazardous drinking might emerge with extended follow-up and increased death count.

Comparisons of effect sizes on cause-specific mortality with and without adjustment for clinical measurements and biomarkers provided additional insight into the complex relationship between alcohol consumption and mortality risks. The observed attenuations in point estimates of HRs in the non-drinking group with additional adjustments for biomedical factors further supports our hypothesis that a poorer health in this group explains the slightly increased mortality. In contrast, the increased HR point estimates after adjusting for biomedical factors for hazardous and harmful drinking groups suggest these groups could be on average healthier than low-risk drinkers, which could be a reason for both high alcohol tolerance and the slightly reduced HRs in models adjusted for socio-demographic factors only.

### Predictors of mortality in narcology patients and the general population

Our study assessed the predictive power of non-communicable disease risk factors and biomarkers, primarily cardiovascular, on mortality risks in the general population (including all drinking groups) and narcology patients, who exhibited higher mortality. This analysis highlighted the limited applicability of widely used sociodemographic and health parameters in Russian clinical practice for risk management in narcology patients.

Notably, we found that higher education was a protective factor in the general population but a risk factor for cardiovascular mortality in narcology patients. This could be due to collider bias. Higher education typically reduces both mortality risk and the likelihood of becoming a narcology patient. However, among those who are narcology patients, individuals with higher education might have specific characteristics that increase their mortality risk, thus masking the protective effect of education. Additionally, this suggests that socioeconomic advantages do not counterbalance the health risks associated with chronic severe drinking. Individuals that are more educated may be more vulnerable to harmful effects of alcohol dependence, potentially due to downward social drift [46, 83]. In contrast, smoking had no significant impact on mortality in narcology patients, suggesting chronic alcohol misuse overshadows smoking risks. Male sex was also a less pronounced cardiovascular risk factor in narcology patients, indicating reduced sex differences in this group [84].

BMI emerged as a significant cardiovascular risk factor exclusively in narcology patients, underscoring the combined effects of heavy drinking and higher BMI on mortality, which may exceed the risks posed by these factors independently [85]. However, BMI’s role as a protective factor against deaths from external causes in the main study was not mirrored in narcology patients, implying that nutritional reserves might mitigate risky behaviors in the general population [86]. No significant differences were noted in neoplasm deaths between groups, likely due to limited cases and insufficient follow-up duration.

Regarding cardiovascular biomarkers, the increased hs-CRP and reduced LVEF were significant mortality predictors in the general population but showed diminished effects in narcology patients. The reduced impact of hs-CRP in narcology patients could relate to baseline twofold higher concentration compared to other the general population. LVEF’s reduced role aligns with chronic severe drinking altering the cardiovascular mortality profile, potentially accelerating progression to acute conditions. HbA1c and Cystatin C, however, consistently predicted all-cause mortality across both populations, indicating their independence from alcohol exposure. NT-proBNP also remained significant mortality predictor in both groups, although with lesser effects in narcology patients.

This study also acknowledges the potential influence of other factors not assessed. In related research, Fuster et al. identified anemia as a significant risk in alcohol-dependent patients [45], while Rivas et al. noted early admission, comorbidities, and methadone treatment as predictors [44]. Liao et al. and Chen et al. found red blood cell distribution width and hyperlactatemia as significant in short-term mortality studies [87, 88]. Pan et al. demonstrated the relevance of plasma anion gap in predicting one-year all-cause mortality in first-admission AUD patients [89]. Future research should consider these elements in risk assessment for severe drinking populations.

### Strengths and limitations

This study’s strength lies in its prospective design and the capacity to adjust for a broad spectrum of health parameters and biomarkers. The study stands among the first to explore mortality predictors in Russian individuals treated for alcohol-related diagnoses. We also obtained mortality data from the Arkhangelsk Regional Mortality Database, which compiles information from both medical death certificates from medical organizations and data from regional civil registration offices. Regular cross-checks between these two sources ensures high data completeness and quality, minimizing the risk of misclassification of death causes.

Our findings should be considered in light of the limitations. Firstly, we conducted a secondary analysis with the baseline data on alcohol consumption, cardiovascular and other parameters available in the Know Your Heart study. Changes in these factors over time were not captured, possibly affecting the accuracy of the assigned drinking categories and their associated risk assessments. The inability to capture dynamic patterns of alcohol consumption and health parameters may introduce bias in understanding their impact on mortality.

Another potential limitation is the use of self-reported alcohol consumption data, which may have low reliability. To address this issue, we applied a multi-tool approach to collect the self-reported data (questions about drinking in the past 12 months, AUDIT test, CAGE test, and questions about specific signs of harmful drinking pattern), partly used at the baseline interview, and partly at the health check. The suitability of our approach was supported by the presented upward trends in GGT and CDT levels along with increasing drinking category.

Focusing on linear relationships of health parameters also possibly distorts the interactive effects of alcohol consumption on mortality risk. The inability to differentiate lifelong abstainers from former drinkers in the non-drinking group introduces a potential bias, possibly explaining the increased mortality rates observed in this group. Additionally, the narcology group is at a generally higher risk overall, making conventional risk factors less predictive in this context.

The study may also exhibit selection bias, evidenced by age-standardized mortality rates per 100,000 among KYH participants being 6% lower in men (1621 vs. 1724) and 18% lower in women (547 vs. 670) compared to the urban adult population of the Arkhangelsk Region [90]. This difference suggests that our cohort might not fully represent the broader population, but we focused on hazard ratios in various alcohol consumption levels, not on the mortality rates, minimizing the impact of this potential bias on the findings. In addition, our study population comprises both a population sample (non-drinking, low-risk drinking, hazardous drinking, and harmful drinking groups) and narcology patients, a specific sample from a clinic. This mixed sampling approach may limit the generalizability of our findings to the general population. However the decision to include a sample of participants in treatment for alcohol-related problems was informed by concerns about under-representation of this group within a population-based survey.

The lower number of deaths among harmful drinking and narcology women did not allow separate analysis by sex. Consequently, our results may have restricted applicability across genders. However, we employed age and sex standardization for mortality rates and adjustments in our models to isolate the effects of alcohol consumption, ensuring a more accurate representation of its impact.

To address the limited follow-up duration, we conducted three analyses at half-year intervals, consistently confirming the stability of our findings. This suggests that longer durations would likely reinforce similar results with narrower dispersion without fundamentally altering our findings. Also, we had no data on loss to follow up and the inability to track deaths outside the Arkhangelsk Region presents a limitation, potentially introducing bias in the mortality data.

These limitations highlight areas for future longitudinal research within diverse populations, analyzing new mortality predictors and non-linear relationships, and capturing dynamic alcohol consumption patterns.

## Conclusion

This study assessed the impact of levels of alcohol consumption on mortality among Russian adults, including those treated for alcohol-related diagnoses (narcology patients). Across all categories, cardiovascular diseases emerged as the primary cause of death, with narcology patients exhibiting a threefold increase in all-cause and cardiovascular mortality and a ninefold increase in mortality from external causes relative to low-risk drinkers. In the general population, factors such as smoking, WHR, resting HR, SBP, and Hs-CRP escalated all-cause mortality risks, while higher education and LVEF were protective. However, these effects were not observed in narcology patients, where higher education and BMI were exclusively risk factors for cardiovascular mortality, and NT-proBNP and LVEF had lower predictive power. The findings indicate that conventional cardiovascular risk factors and biomarkers have limited predictive value in narcology patients, suggesting the need for a broader approach to health risk management in this population.

## Electronic supplementary material

Below is the link to the electronic supplementary material.

Supplementary Material 1

## Data Availability

Data from the Know Your Heart Study are available from the authors upon reasonable request with permission of the Know Your Heart Steering Group (https://metadata.knowyourheart.science/).
