# Supplementary material for: Alcohol and cause-specific mortality in Russia: the Know Your Heart Study 2015–23
Source: BMC Public Health. 2024 Nov 12;24:3128. doi: 10.1186/s12889-024-20674-8 (PMC11555830; doi:10.1186/s12889-024-20674-8)
Supplement: Supplementary file 1 — Supplementary Material 1 [file 12889_2024_20674_MOESM1_ESM.docx]

**Supplementary Table 1.** Deaths by alcohol consumption levels and ICD-10 classes of underlying causes of death, N (%)

| ICD-10 Class | Levels of alcohol consumption | | | | | Total |
| --- | --- | --- | --- | --- | --- | --- |
|  | Low-risk | Non-drinkers | Hazardous | Harmful | Narcology |  |
| N (%) | 88 (39.5%) | 23 (10.3%) | 23 (10.3%) | 16 (7.2%) | 73 (32.7%) | 223 (100.0%) |
| Diseases of the circulatory system (I00-I99) | 32 (36.4%) | 10 (43.5%) | 10 (43.5%) | 7 (43.8%) | 25 (34.2%) | 84 (37.7%) |
| Neoplasms (C00-D48) | 28 (31.8%) | 6 (26.1%) | 5 (21.7%) | < 5* | < 5* | 45 (20.2%) |
| External causes of morbidity and mortality (V01-Y98) | 5 (5.7%) | < 5* | < 5* | 4 (25.0%) | 20 (27.4%) | 31 (13.9%) |
| Certain infectious and parasitic diseases (A00-B99) | 9 (10.2%) | < 5* | < 5* | / | < 5* | 14 (6.3%) |
| Diseases of the digestive system (K00-K93) | 4 (4.5%) | / | < 5* | / | 7 (9.6%) | 13 (5.8%) |
| Diseases of the respiratory system (J00-J99) | 5 (5.7%) | / | / | / | 5 (6.8%) | 10 (4.5%) |
| Symptoms, signs and abnormal clinical and laboratory findings, not elsewhere classified (R00-R99) | < 5* | / | < 5* | < 5* | 5 (6.8%) | 9 (4.0%) |
| Endocrine, nutritional and metabolic diseases (E00-E90) | < 5* | < 5* | < 5* | / | / | 7 (3.1%) |
| Diseases of the nervous system (G00-G99) | / | < 5* | < 5* | < 5* | < 5* | 7 (3.1%) |
| Diseases of the genitourinary system (N00-N99) | < 5* | / | / | / | < 5* | < 5* |
| Congenital malformations, deformations and chromosomal abnormalities (Q00-Q99) | / | / | < 5* | / | / | < 5* |
| * Numbers redacted due to cell counts less than 5 to preserve data anonymity. | | | | | | |
